# Development and validation of the tiredness identification index: a combined scale for the assessment of fatigue and sleepiness

**DOI:** 10.1093/sleepadvances/zpag072

**Published:** 2026-07-02

**Authors:** Sarah Reeve, Bryony Sheaves, Bao Sheng Loe, Daniel Freeman

**Affiliations:** Norwich Medical School, University of East Anglia, Norwich, United Kingdom; Cambridgeshire and Peterborough NHS Foundation Trust, Cambridge, United Kingdom; Department of Experimental Psychology, University of Oxford, Oxford, United Kingdom; Oxford Health NHS Foundation Trust, Oxford, United Kingdom; Cambridge Psychometrics Centre, University of Cambridge, Cambridge, United Kingdom; Department of Experimental Psychology, University of Oxford, Oxford, United Kingdom; Oxford Health NHS Foundation Trust, Oxford, United Kingdom

**Keywords:** sleepiness, fatigue, tiredness, measurement

## Abstract

**Study Objectives:**

Tiredness is a common presentation composed of sleepiness (propensity towards sleeping) and fatigue (feelings of physiological exhaustion). Differentiating between these presentations is important for diagnosis and management. The current study reports on the development and validation of an integrated measure of tiredness (Tiredness Identification Index—TIDI) incorporating assessment of sleepiness and fatigue, and the relationship of the measure with sleep and mental health outcomes.

**Methods:**

A sample of 6706 adult participants responded to a pool of sleepiness and fatigue items. Regularized structural equation modelling and confirmatory factor analysis were used to refine the items and factor structure based on convergence against validation measures of fatigue and sleepiness. Regression analyses were then applied to test relationships of the sleepiness and fatigue factors with sleep, mental health, and wellbeing variables.

**Results:**

An 8-item scale was developed, composed of a sleepiness (TIDI-sleepiness) and fatigue (TIDI-fatigue) factor with divergent and convergent validity, alongside a general tiredness factor. Higher TIDI-sleepiness was associated with increased apnoea risk and shorter sleep duration, whereas TIDI-fatigue was associated with increased insomnia and reduced sleep efficiency. Controlling for TIDI-sleepiness, TIDI-fatigue was associated with increased depression, anxiety, reduced well-being, and lower physical activity. Increased TIDI-sleepiness and TIDI-fatigue were both associated with paranoia and hallucinations.

**Conclusions:**

The results indicate that the TIDI can be used as a brief scale to measure and differentiate fatigue and sleepiness, particularly in non-clinical populations and for research purposes. Assessment of its validity in discriminating fatigue and sleepiness in relevant clinical sleep, mental, and physical health populations should be a focus of future research.

**Statement of Significance**

Tiredness is a common concern that is linked to a wide range of sleep-related, physical health, and mental health issues. Tiredness is understood to reflect sleepiness (i.e. wanting to go to sleep) or fatigue (i.e. physical exhaustion) or both, but assessment has been limited by difficulty in discriminating sleepiness and fatigue. This paper reports a new measure that enables discrimination between sleepiness and fatigue, and its application to testing relationships between fatigue, sleepiness, sleep, and mental health in a large non-clinical population. The measure requires further testing and validation in the broad range of clinical populations for which fatigue and sleepiness are concerns, but holds the potential to improve the assessment and understanding of fatigue, sleepiness, and tiredness.

## Graphical Abstract

**Graphical Abstract:**
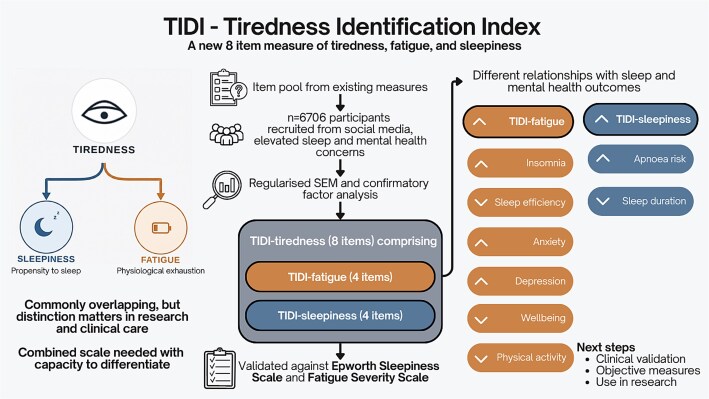

One of the most common concerns expressed across physical and mental health patient populations is tiredness [1]. This has historically been broken down into two distinct phenomena, those of fatigue and sleepiness [2, 3]. In non-clinical populations fatigue typically reflects a physiological signal that rest is required (with or without sleeping), and is normally the result of energy being expended (e.g. after exercise). Sleepiness is often described as a “behavioural propensity towards sleep” which typically requires resolution by sleeping [4, 5]. In non-clinical populations fatigue and sleepiness may often co-occur e.g. at the end of the day when the circadian drive for arousal is low and homeostatic sleep pressure is high [5, 6], and when energy would have been expended. There is an acknowledged difficulty in discriminating between tiredness, sleepiness, and fatigue given that the terms are near-synonymous in common usage [4], and there is a lack of appropriate assessment tools to allow accurate discrimination between these presentations [5, 7].

The underlying mechanisms of fatigue and sleepiness are complex and needing further investigation but are differentiable. Sleepiness is mostly linked with a reduction in inhibition of sleep-promoting systems in the brain [8]—for example, the orexin system which is disrupted in narcolepsy, leading to sudden sleep attacks [9]. In contrast, mechanisms underlying fatigue are typically proposed to act across the whole body, with mitochondrial and inflammation-based hypotheses prominent in the current literature [10]. At the diagnostic level, fatigue and sleepiness may differentiate different sleep disorder indications—for example, insomnia is more strongly associated with fatigue and sleep apnoea with sleepiness [7, 11–13]—and therefore differential indicators could assist with discriminating these common conditions. As well as the importance of discriminating between fatigue and sleepiness, better accounting for their overlap would facilitate research addressed at conditions that involve sleepiness and/or fatigue as core elements e.g. chronic fatigue syndrome [14–16].

Fatigue and sleepiness are both common alongside mental health problems, and may be linked to worsened symptoms and functioning. Fatigue is strongly associated with depression [17]. High levels of sleepiness (hypersomnia) are common in mood disorders and may be linked to a more severe clinical profile [18, 19]. In psychosis fatigue is commonly reported [20], and sleepiness is also often problematic [21]. These symptoms are often ascribed to sedation from medication, but could also be linked to high rates of sleep disorders and low daytime activity [21, 22].

There are several widely used fatigue scales, but in some cases these incorporate items that may index sleepiness (e.g. Chalder Fatigue Scale [23] item 3: “do you feel sleepy or drowsy?”). With respect to sleepiness there are also several existing measures, the most commonly used being the Epworth Sleepiness Scale (ESS) which asks patients to rate their likelihood of falling asleep in daily situations. However its validity in populations with severe mental health problems may be limited [24] because patients may be less likely to engage with the situations listed (e.g. sitting in a public place such as a cinema or a meeting). Only two measures were identified that assess sleepiness and fatigue in the same measure: the Sleep Wake Activity Inventory [25]and the Adjective Checklist to measure fatigue, anergy, consciousness, energized, sleepiness [26], neither of which has been widely adopted, potentially due to their significant length (both ≥50 items).

The aim of this study was to: (1) develop a novel and brief scale of sleepiness and fatigue (the Tiredness Identification Index (TIDI); (2) assess the TIDI’s psychometric properties; and (3) assess the relationship between the TIDI and sleep features, sleep disorders, mental health symptoms, physical activity, and quality of life in a general population sample.

## Materials and methods

### Study design and ethical approval

A questionnaire development study was conducted using an online cross-sectional self-report survey. The study was reviewed and approved by the University of Oxford Central Ethics Committee (ref: R57924/RE001).

### Recruitment

Recruitment was carried out via a social media platform (Facebook). A study specific page with university branding was set up, from which the study was advertised. Paid promotion of posts was used to advertise the study to eligible participants. Participants completing the study were offered entry into a prize draw to win shopping vouchers. Eligible adults were those aged 18 and over from the United Kingdom, with no further inclusion or exclusion criteria for participation. 9049 individuals consented to take part. Data collection was carried out between 29th June 2018 and 27th August 2018.

Participants who were aged under 18 (n = 12) or who completed the survey in less than 5 minutes (n = 15) were excluded from the dataset, followed by participants who had not completed the minimum dataset i.e. demographic information, the TIDI item pool, and the validation measures (n = 2286). This resulted in 6736 participants in the validation dataset.

### Measures

Tiredness Identification Index item pool: An initial item pool (n = 40; see Supplementary Materials 1) was developed. Items were derived from pooling existing fatigue and sleepiness measures, with adaptations to improve consistency including removing specific situational contexts (e.g. SWAI [25] item 29: “I get drowsy driving for a few minutes” became “Feeling drowsy”); tense and response mode (e.g. ESS [27] items assessing conditional likelihood of dozing became “Feeling dozy”), and general simplification (e.g. FSS [28] item “My motivation is lower when I am fatigued” became “Lacking motivation”). Additional items were also identified from behavioural studies [29] e.g. “Eyes feeling heavy”. Several further sleepiness items (e.g. “Eyes feeling itchy or stingy”) were generated by the study team to balance a preponderance of fatigue items in the item pool. Items from existing scales that related to the impact of sleepiness or fatigue rather than sleepiness or fatigue itself (e.g. “Being easily irritated”) were included in the item pool to allow for any potential discriminatory capacity between sleepiness and fatigue. The item derivation, refinement, and generation process was carried out by clinical psychologists with clinical experience of treating sleepiness and fatigue (BS and DF), and a post-doctoral researcher specializing in understanding sleep disruption (SR), with all agreeing on the final item pool.

Items were rated for the past week on a Likert scale from “0 (Not at all)” to “4 (Extreme)”, with the instruction ‘This questionnaire assesses the impact of tiredness. In the last week, how much of an issue have you had with…”. A severity response mode was chosen for the scale, as this was considered to be more clinically relevant (by virtue of assessing distress and disruption) than a frequency scale. The introduction and title of the scale references ‘tiredness’ to exploit the double meaning of tiredness—fatigue and/or sleepy—in English.

Demographic information: Participants were asked to self-report their age, gender, ethnicity, occupational status, and education level. Participants self-reported experiences of mental health difficulties, their diagnosis, whether the difficulties were ongoing, and if they were currently taking medication for their mental health.

Validation measures: The ESS [27] was used as a measure of convergent validity for sleepiness and divergent validity for fatigue. In the ESS respondents rate their likelihood of falling asleep in a variety of situations on a 0-3 scale where 0 indicates no chance and 3 represents a high chance of dozing. These responses are totaled (range 0–24) with higher scores reflecting greater sleepiness. Consistent with scale guidelines, we adopted a cut-off score of ≥10 to indicate problematic sleepiness.

The Fatigue Severity Scale (FSS) [30] was selected as the measure for convergent validity for fatigue and divergent validity for sleepiness. This is a self-report questionnaire that assesses the severity of fatigue in various aspects of life. Each item is rated from 1 (strongly disagree) to 7 (strongly agree), with higher scores indicating more severe fatigue. The FSS score is the average of these responses, for which a score ≥4 indicates problematic fatigue.

Sleep measures: Participants were asked questions about their sleep timing based on “the majority of days” including time in to bed, time trying to start to sleep, sleep onset latency, night waking, sleep offset, and time out of bed, based on the consensus sleep diary [31]. This allowed estimation of average sleep duration and sleep efficiency (i.e. percentage of time in bed spent asleep). Participants were also asked to report nap frequency and duration.

Sleep disorder symptoms were measured using (1) the Insomnia Severity Index (ISI) [32], a 7-item scale measuring symptoms of insomnia that can be used dimensionally or with a cut off of ≥15 indicating presence of clinically significant insomnia; and (2) the STOP-BANG screening measure for sleep apnoea [33]. A score ≥5 indicates a high likelihood of obstructive sleep apnoea being present. Participants were also asked to report how many nightmares they had had over the previous two weeks.

Mental health and general wellbeing: Depression and anxiety were assessed utilizing the relevant subscales of the shortened Depression Anxiety and Stress Scale [34], each seven items on a 0–3 severity response scale with higher scores indicating higher severity.

Paranoia and hallucinations were assessed using the respective subscales of the Specific Psychotic Experiences Scale (SPEQ) [35]. 15 items assessed paranoia, nine items assessed hallucinations, each using a 0-5 frequency response scale. Higher scores indicate higher severity of symptoms.

The 14-item Warwick Edinburgh Mental Wellbeing Scale (WEMWBS) [36] was used to index positive mental wellbeing. Each item is rated on a 1-5 scale from “None of the time” to “All of the time” with greater scores indicating higher levels of wellbeing (range 14–70). The top 15% of wellbeing scores range from 60 to 70, and the bottom 15% between 14 and 42 [36].

Levels of physical activity were assessed using the short form (7-item) International Physical Activity Questionnaire (IPAQ) [37] which assesses time spent doing different intensity levels of exercise over the previous week. The scale has established cut-offs, and provides categorization of participants into low, medium, and high levels of physical activity accordingly.

### Analysis

Analyses were conducted in R, version 4.2.2, utilizing lavaan [38], psych [39], regsem [40] and pROC [41] packages. The analysis script is available on OSF (https://osf.io/pvk25/).

Scale development: To assist with initial scale development the data were split with 75% of responses (n = 5052) allocated to a training dataset, and 25% towards a test dataset (n = 1684). Based on our aim to develop an integrated measure of fatigue and sleepiness, we used the training dataset to conduct a regularized structural equation modelling (RegSEM) lasso analysis to identify individual items that predicted sleepiness and/or fatigue based on the validation measures (the ESS and FSS). RegSEM lasso is specifically designed to allow for the assessment of a complex model with a large number of parameters but with a limited number of observations [42]. This is accomplished by simplifying the measurement model and retaining only important predictors—in this case those that load onto the ESS and FSS—by applying penalties to the regression paths to drive their coefficient towards zero, thereby reducing the item pool. Items with a beta coefficient > 0.02 (indicating at least some relationship) on FSS or ESS were retained. Retained items from the RegSEM analysis were factor analyzed using minimum residual estimation to further reduce the item pool.

Given the pre-specified theoretically driven factor structure, CFA was applied to remove further items by assessing modification indices, factor loadings (<0.4 cut-off), item content overlap, and an aim for parsimony and balance between the sleepiness and fatigue factors. We employed the robust maximum likelihood estimator to account for data skewness. Model fit was determined using recommended thresholds of above 0.95 on the comparative fit index (CFI) and Tucker–Lewis index (TLI), below 0.06 on the root mean square of approximation (RMSEA), and below 0.08 on the standardized root mean square residual (SRMR) [43]. The final scale was then validated on the test dataset to confirm its factorial structure. Appropriateness of a bifactor model (with a higher order “tiredness” factor) was also undertaken to assess the appropriateness of calculating a total score.

Validation: Convergent and divergent validity was also evaluated by testing model fit of the identified sleepiness and fatigue subscales against the ESS versus the FSS, and receiver operating curve (ROC) analysis, including sensitivity and specificity of convergent and divergent cut offs for the subscales against the validation measures.

Measurement invariance: To evaluate whether the TIDI operated equivalently across gender, a series of multi-group confirmatory factor analyses tests were conducted for configural, metric, and scalar invariance. Model fit was assessed using χ^2^ difference tests alongside changes in approximate fit indices (CFI, RMSEA, and SRMR), with threshold change criteria for invariance applied based on criteria (ΔCFI ≤ .01, ΔRMSEA ≤ .015, ΔSRMR ≤ 0.015; Chen, 2007).

Relationships with sleep, mental health, and wellbeing: Linear regression analyses were conducted to explore: (1) the relationship between TIDI total score (tiredness) with individual sleep, mental health, and wellbeing variables and (2) the relationship between both TIDI subscales (fatigue and sleepiness) and individual sleep, mental health, and wellbeing variables. In the latter case both fatigue and sleepiness were included in the model to attempt to control for their known overlap. Individual regressions (fatigue or sleepiness and outcome measures) were also carried out. 

## Results

### Scale development and validation

Demographic and clinical information: Respondents reported an average age of 42.0 years (SD = 15.8), with the majority being female (n = 5161, 76.7%). The majority of respondents (n = 6418, 95.2%) reported their ethnicity as White (any background), had at least an undergraduate degree, and were part- or full-time employed. See Table 1 for full demographic information.

**Table 1:** Demographics of participant group

**Demographics**	**Statistic**
Age (mean, SD)	42.0 (15.8)
Gender (n, %)	
Female	5161 (76.7)
Male	1472 (21.9)
Other or prefer not to say	103 (1.6)
Ethnicity (n, %)	
White (any background)	6418 (95.2)
Black (any background)	11 (0.2)
Asian (any background)	97 (1.4)
Mixed or multiple ethnic groups	131 (1.9)
Other or prefer not to say	81 (1.2)
Highest educational level achieved (n, %)	
High/secondary school or equivalent	726 (10.8)
Sixth form college or equivalent	1309 (19.5)
Higher education diploma	858 (12.7)
Undergraduate degree	2107 (31.3)
Post-graduate degree	1730 (25.7)
Employment (n, %)	
Unemployed and not in education	436 (6.8)
Student	994 (14.8)
Homemaker or carer	324 (4.8)
Part time employed	1199 (17.8)
Full-time employed	2955 (43.9)
Retired	825 (12.3)
Mental health history (n, %)	
History of mental health problem	4465 (66.3)
Current mental health problem	2871 (42.5)
Taking medication for mental health problem	1815 (26.9)

Two-thirds of participants (n = 4465, 66.3%) reported having experienced mental health difficulties, of which the overwhelmingly most common were depression and/or anxiety (4245 participants, 95.1% of those reporting mental health difficulties). 2871 participants described their difficulties as current (42.6% of those reporting mental health difficulties), and 1815 (40.6% of those reporting mental health difficulties, 26.9% of the whole sample).

#### Descriptive data for participant group—sleep and mental health

Table 2 reports descriptive statistics for the study participants. Participants in the study reported adequate average sleep duration (7 hours and 6 minutes) but relatively low sleep efficiency at 78% average (85% or above being considered normative) [44]. 37.5% of participants scored above threshold for problematic sleepiness on the ESS, and 51.3% scored above the cut-off for problematic fatigue on the FSS. ISI scores in the group were high, with 2623 respondents (41.0%) scoring above the moderate insomnia threshold (≥15). 1917 participants reported napping during the day (27.6%). Of the participants who completed the STOP-BANG almost 19.4% scored at high risk. 40.5% (n = 2665) of responders reported at least weekly nightmares.

**Table 2:** Descriptive statistics for sleep, mental health and wellbeing variables

**Measure**	**Mean (SD)**	**Completed, n (%)** ^*^
**Sleep**		
Sleepiness (ESS)	9.12 (4.8)	6736 (100)
Above cut off (≥11 total) (n, %)	2528 (37.5)	
Fatigue (FSS)	36.4 (13.8)	6736 (100)
Above cut-off (≥4 average score) (n, %)	3455 (51.3)	
Sleep diary		6494 (96.4)
Sleep duration (mins)	426 (102.0)	
Sleep efficiency	0.78 (0.2)	
Reporting naps (n, %)	1917 (27.6)	6633 (98.4)
Of those napping, average duration (mins)	66.65 (46.2)	
Insomnia Severity Index	13.83 (5.9)	6554 (97.3)
Above cut off (≥15 total) (n, %)	2623 (41.0)	
STOP-BANG^**^	1.45 (1.4)	3998 (59.4)
Above cut off (≥3) (n, %)	774 (19.4%)	
Nightmares over past two weeks	1.80 (2.8)	6578 (97.7)
No nightmares	2975 (45.2)	
One nightmare	938 (14.3)	
Two or more nightmares	2665 (40.5)	
**Mental health and wellbeing**		
DASS (mean, SD)		6041 (89.7)
Depression	15.17 (5.7)	
Anxiety	12.58 (4.0)	
SPEQ (mean, SD)		5997 (89.0)
Paranoia	12.6 (13.5)	
Endorsing at least one item (n, %)	4864 (82.8)	
Hallucinations	4.66 (6.4)	
Endorsing at least one item (n, %)	3612 (60.2)	
Wellbeing (WEMWBS) (mean, SD)	40.40 (10.0)	5927 (88.0)
Physical activity (IPAQ)		5806 (86.2)
Minutes of physical activity (mean, SD)	2480 (2751.1)	
Low activity (n, %)	749 (12.9)	
Moderate activity (n, %)	2870 (49.4)	
High activity (n, %)	2187 (37.7)	

^*^Total n – 6736; attrition across all measures except STOP-BANG were due to participants partially completing the study; Any % calculations use the n completed as denominator.

^**^STOP-BANG was optional due to reliance on knowing BMI

Participants reported a generally high level of mental health symptomatology. Relatively high proportions endorsed at least one paranoia symptom (82.7%) and hallucinatory experiences were also relatively common (60.2% of the participants reporting at least one). The average WEMWBS score was in the “low wellbeing” range (<42) i.e. bottom 15% of the normative population.

#### Scale development

Bartlett’s test for Sphericity confirmed that the item correlations were appropriate for conducting an EFA (χ^2^ = 153 421, df = 780, *p* < .001). The Kaiser-Meyer-Olkin value was in the “marvellous” range (overall KMO = 0.98, all items KMO > 0.94) indicating adequate sampling for factor analysis.

The CFA was carried out on the test dataset (n = 1684). The bifactor model (robust χ^2^(12), *p* < .001, SRMR = 0.023, CFI = 0.98, TLI-0.96, RMSEA = 0.07) represented a marginal improvement on the two-factor model in terms of model fit (robust χ^2^(19) *p* < .001, SRMR = 0.04, CFI = 0.95, TLI = 0.93, RMSEA = 0.09) and fits with the proposed theoretical structure of sleepiness and fatigue as components of tiredness. Internal consistency of the sleepiness (Cronbach’s alpha = 0.79), fatigue (Cronbach’s alpha = 0.88), and total factors (Cronbach’s alpha = 0.89) were all in the acceptable or good range. Factor loadings are available in Supplementary Materials. See Table 3 for the TIDI final scale, with a printable version included in Supplementary Materials.

**Table 3:** Tiredness identification index (TIDI)

**This questionnaire assesses the impact of tiredness. In the last week, how much of an issue have you had with….**					
	**0 (none)**	**1 (slight)**	**2 (moderate)**	**3 (very much)**	**4 (extreme)**
**Feeling unable to concentrate**	0	1	2	3	4
**Keeping eyes open**	0	1	2	3	4
**Blinking more than usual**	0	1	2	3	4
**Feeling weak**	0	1	2	3	4
**Feeling low in energy**	0	1	2	3	4
**Wanting to nap**	0	1	2	3	4
**Drifting off**	0	1	2	3	4
**Feeling exhausted**	0	1	2	3	4

Items 2, 3, 6, 7 = sleepiness subscale (TIDI-s)

Items 1, 4, 5, 8 = fatigue subscale (TIDI-f)

#### Measurement invariance

Measurement invariance was predominantly supported with respect to gender (which was tested excluding non-binary or “prefer not to say” respondents due to a low sample size of n = 103, 1.6%). The configural model provided an excellent fit to the data (RMSEA = 0.067, SRMR 0.019, CFI 0.990, TLI 0.969), supporting that the factor structure applied similarly across gender groups. The metric invariance model was also an excellent fit to the data (RMSEA = 0.050, SRMR = 0.021, CFI = 0.990, TLI = 0.982), with the chi-square difference test between the metric and configural model being non-significant. Differences in fit relative to the configural model were largely within bounds (ΔCFI = 0.00, ΔSRMR = 0.002), however the ΔRMSEA (0.017) was slightly outside of the acceptable bounds of 0.015. The scalar model was also a substantial fit to the data (RMSEA = 0.054, SRMR = 0.023, CFI = 0.987, TLI = 0.980), and while the chi-square difference test between the scalar and metric model was significant, there were minimal differences in fit detected (ΔCFI = 0.003, ΔRMSEA = 0.004, ΔSRMS = 0.002).

Measurement invariance was also largely supported with respect to current mental health status. Both the configural (RMSEA = 0.066, SRMR = 0.020, CFI = 0.989, TLI = 0.965) and metric invariance models indicated excellent fit to the data (RMSEA = 0.050, SRMR = 0.023, CFI = 0.989, TLI = 0.979). The chi-square difference test between the configural and metric models was non-significant, and the differences in fit between the two models were largely bounds (ΔCFI =0.00, ΔSRMR = 0.003), with the exception of the RMSEA index (ΔRMSEA = 0.016). The scalar model supported invariance with respect to the intercept with good model fit (RMSEA = 0.050, SRMR = 0.024, CFI = 0.987, TLI = 0.980), and while the chi-square test indicated significant differences, all changes from the metric model were within accepted bounds (ΔCFI = 0.002, ΔRMSEA = 0.000, ΔSRMS = 0.001).

#### Validation

Divergent validity was tested both by comparing convergent (e.g. ESS correlated with sleepiness subscale) and divergent subscale models (e.g. ESS correlated with the fatigue subscale) with respect to variance explained, and by fitting convergent and divergent ROC curves. With respect to the convergent model, the sleepiness scale explained 58% of the variance of the ESS score and the fatigue scale 63% of the FSS within the test sample. When the reverse was tested, the sleepiness scale explained 34% of the FSS score variance, and the fatigue scale explained 22% of the variance in the ESS scores.

A ROC analysis indicated that a threshold of 4 on the sleepiness subscale predicted being above the ESS cut-off (≥10) with a sensitivity of 74.9 (95%CI 73.8–76.0) and specificity of 76.5 (95%CI 72.6–79.8) with an AUC of 0.827. A threshold of 8 on the fatigue subscale predicted being above the FSS cut off (≥4) with a sensitivity of 75.6% (95% CI 74.1–77.0) and a specificity of 78.6% (95% CI 77.2–80.0) with an AUC of 0.852.

#### Relationship between TIDI and demographic variables

There was a significant small negative correlation between age and TIDI-total scores (r = -0.09, *p* = .02). Males reported a significantly lower average TIDI-total score (11.87, SD = 6.6) than females (13.53, SD = 6.8) (t = -9,4, df = 2435.4, *p* < .001, Cohen’s d = 0.25). No significant differences in TIDI-total score were found by ethnicity (F (5) = 1.79, *p* = .109).

Significant differences were found between education groups and TIDI-total scores, with increasing levels of education associated with reduced TIDI-total score (F (5) = 32.45, *p* < .001). Significant differences were found between employment groups (F (5) = 49.53, *p* < .001), with inspection of results indicating higher TIDI-total scores in unemployed and homemaker groups, and lower TIDI scores in retired, students, part-time and full-time employed respondents.

Respondents with current mental health issues reported higher average TIDI-total scores (15.93, SD = 6.4) than those not reporting a current mental health problem (11.74, SD = 6.3) t = 20.59, df = 2937, *p* < .001, d = 0.66). Among participants reporting a current mental health problem, those reporting taking prescribed medication reported higher TIDI-total scores (16.5, SD = 6.5) than those not (13.1, SD = 6.3; t = 17.26, df = 3794, *p* < .001, d = 0.53).

### Relationship of the TIDI with sleep, mental health, and wellbeing measures

#### Sleep measures

Results are reported in Table 4. Increased TIDI-total score was significantly associated with decreased sleep duration, decreased sleep efficiency, increased nap duration, increased insomnia, increased nightmare frequency, and increased scores on the STOP-BANG apnoea risk index. The largest association of the total TIDI score was with insomnia (β = 0.56), with other relationships mostly moderate in size (|β| between 0.18 and 0.27), except for sleep duration (β = -0.04) which had a small association.

**Table 4:** Regressions between TIDI total and TIDI subscale scores with outcome measures

**Measure**	**Regression with TIDI total score**					**Regression with TIDI subscale scores**				
	**Predictor**	**Estimate (StdError)**	**Standardised estimate (StdError)**	**Statistic**	***p*-value**	**Predictor**	**Estimate (StdError)**	**Standardised estimate (StdError)**	**Statistic**	***p*-value**
**Sleep**										
Sleep duration (mins)	(Intercept)	434.55 (3.77)	0.00 (0.02)	−0.01	0.989	(Intercept)	433.52 (3.81)	0.00 (0.02)	−0.01	0.993
	**TIDI_t**	**−0.56 (0.24)**	**−0.04 (0.02)**	**−2.36**	**0.018**	TIDI_f	0.27 (0.54)	0.01 (0.02)	0.51	0.612
						**TIDI_s**	**−1.51 (0.6)**	**−0.05 (0.02)**	**−2.50**	**0.012**
Sleep efficiency	(Intercept)	0.84 (0.01)	0.00 (0.02)	−0.07	0.946	(Intercept)	0.85 (0.01)	0.00 (0.02)	−0.08	0.934
	**TIDI_t**	**0.00 (0.00)**	**−0.18 (0.02)**	**−11.80**	**<0.001**	**TIDI_f**	**−0.01 (0)**	**−0.21 (0.02)**	**−10.78**	**<0.001**
						TIDI_s	0.00 (0.0)	0.02 (0.02)	1.08	0.281
Nap duration (mins)	(Intercept)	33.76 (3.57)	−0.13 (0.03)	−4.46	<0.001	(Intercept)	33.7 (3.55)	−0.10 (0.03)	−3.39	0.001
	**TIDI_t**	**1.88 (0.19)**	**0.27 (0.03)**	**9.84**	**<0.001**	**TIDI_f**	**3.42 (0.4)**	**0.29 (0.03)**	**8.46**	**<0.001**
						TIDI_s	0.01 (0.47)	0.00 (0.04)	0.03	0.977
Insomnia Severity Index (ISI)	(Intercept)	13.62 (0.18)	0.00 (0.01)	−0.17	0.862	(Intercept)	13.22 (0.18)	0.00 (0.01)	−0.18	0.855
	**TIDI_t**	**0.50 (0.01)**	**0.56 (0.01)**	**43.90**	**<0.001**	**TIDI_f**	**0.81 (0.03)**	**0.53 (0.02)**	**32.14**	**<0.001**
						**TIDI_s**	**0.14 (0.03)**	**0.08 (0.02)**	**4.87**	**<0.001**
Nightmares (n over previous two weeks)	(Intercept)	0.65 (0.12)	0.00 (0.02)	−0.06	0.951	(Intercept)	0.54 (0.12)	0.00 (0.02)	−0.08	0.938
	**TIDI_t**	**0.11 (0.01)**	**0.23 (0.02)**	**15.01**	**<0.001**	**TIDI_f**	**0.19 (0.02)**	**0.24 (0.02)**	**11.59**	**<0.001**
						TIDI_s	0.02 (0.02)	0.02 (0.02)	0.84	0.402
STOP-BANG	(Intercept)	0.8× (0.06)	0.01 (0.02)	0.69	0.491	(Intercept)	0.85 (0.06)	0.01 (0.02)	0.64	0.520
	**TIDI_t**	**0.05 (0,00)**	**0.23 (0.02)**	**11.99**	**<0.001**	TIDI_f	0.01 (0.01)	0.02 (0.03)	0.70	0.487
						**TIDI_s**	**0.10 (0.01)**	**0.23 (0.03)**	**9.22**	**<0.001**
**Mental health**										
Depression (DASS)	(Intercept)	9.05 (0.19)	0.00 (0.01)	−0.11	0.915	(Intercept)	8.49 (0.18)	0.00 (0.01)	−0.25	0.806
	**TIDI_t**	**0.43 (0.01)**	**0.50 (0.01)**	**36.11**	**<0.001**	**TIDI_f**	**0.84 (0.03)**	**0.57 (0.02)**	**32.63**	**<0.001**
						TIDI_s	−0.05 (0.03)	−0.03 (0.02)	−1.79	0.073
Anxiety (DASS)	(Intercept)	8.61 (0.14)	0.00 (0.01)	−0.08	0.937	(Intercept)	8.33 (0.14)	0.00 (0.01)	−0.17	0.865
	**TIDI_t**	**0.28 (0.01)**	**0.46 (0.01)**	**32.38**	**<0.001**	**TIDI_f**	**0.48 (0.02)**	**0.47 (0.02)**	**25.35**	**<0.001**
						TIDI_s	0.04 (0.02)	0.03 (0.02)	1.81	0.070
Paranoia (SPEQ)	(Intercept)	19.2 (0.52)	0.00 (0.01)	0.00	1.000	(Intercept)	18.75 (0.53)	0.00 (0.01)	−0.04	0.966
	**TIDI_t**	**0.76 (0.03)**	**0.35 (0.01)**	**23.34**	**<0.001**	**TIDI_f**	**1.11 (0.07)**	**0.29 (0.02)**	**14.97**	**<0.001**
						**TIDI_s**	**0.37 (0.08)**	**0.09 (0.02)**	**4.42**	**<0.001**
Hallucinations (SPEQ)	(Intercept)	0.03 (0.23)	0.00 (0.02)	0.02	0.982	(Intercept)	−0.09 (0.23)	0.00 (0.02)	0.00	0.999
	**TIDI_t**	**0.32 (0.01)**	**0.34 (0.02)**	**22.28**	**<0.001**	**TIDI_f**	**0.41 (0.03)**	**0.25 (0.02)**	**12.46**	**<0.001**
						**TIDI_s**	**0.22 (0.04)**	**0.12 (0.02)**	**6.03**	**<0.001**
Wellbeing (WEMWBS)	(Intercept)	51.05 (0.34)	0.00 (0.01)	−0.03	0.977	(Intercept)	51.94 (0.33)	0.00 (0.01)	0.06	0.956
	**TIDI_t**	**−0.74 (0.02)**	**−0.49 (0.01)**	**−34.93**	**<0.001**	**TIDI_f**	**−1.40 (0.05)**	**−0.54 (0.02)**	**−30.03**	**<0.001**
						TIDI_s	0.02 (0.05)	0.01 (0.02)	0.36	0.717
Physical activity (IPAQ)	(Intercept)	2714.26 (106.77)	0.00 (0.02)	−0.01	0.993	(Intercept)	2764.94 (108.2)	0.00 (0.02)	0.01	0.989
	**TIDI_t**	**−16.19 (6.7×)**	**−0.04 (0.02)**	**−2.42**	**0.016**	**TIDI_f**	**−54.40 (15.21)**	**−0.08 (0.02)**	**−3.58**	**<0.001**
						TIDI_s	27.48 (16.98)	0.03 (0.02)	1.62	0.106

^*^ *p* < 0.001 (threshold adjusted using Bonferonni corrections, n = 40 tests). TIDI_t = TIDI total score, TIDI_f = fatigue, TIDI_s = sleepiness

Repeating the analyses with the separate TIDI subscales entered together indicated that the majority of these relationships were driven by fatigue. TIDI-fatigue was particularly strongly associated with insomnia (β = 0.53) compared with TIDI-sleepiness (β =0.08), although both were significantly associated. For other sleep variables (sleep efficiency – β = -0.21, nap duration – β = 0.29, and nightmares – β = 0.24) TIDI-fatigue was the only significant relationship, with the size of the association being comparable to the TIDI-total score. However, TIDI-sleepiness (but not TIDI-fatigue) was significantly associated with increased apnoea risk (β =0.23) and with reduced sleep duration (β = -0.05).

When tested individually sleepiness and fatigue were significantly associated with all sleep variables (see Supplementary Materials).

#### Mental health and wellbeing measures

Higher TIDI-total score was significantly associated with more severe mental health scores on all measures tested. It was also significantly associated with lower wellbeing and reduced time spent in physical exercise. The size of the associations was mainly in the moderate range (|β| between 0.34 and 0.50), apart from physical activity which was small (β = -0.04).

Repeating the analyses for the mental health measures with both TIDI subscales entered together revealed several differences with respect to contributions of fatigue and sleepiness. TIDI-fatigue (but not TIDI-sleepiness) was associated with increased depression (β = 0.57), anxiety (β = 0.47), reduced wellbeing (β = -0.54), and reduced physical activity (β = -0.08). With respect to psychotic experiences, both TIDI-sleepiness and TIDI-fatigue were associated with increased paranoia (TIDI-sleepiness β = 0.09, TIDI-fatigue β = 0.29) and increased hallucinations (TIDI-sleepiness β = 0.12, TIDI-fatigue β = 0.25). however in both cases TIDI-fatigue was more strongly associated than TIDI sleepiness with the outcomes. TIDI-sleepiness was not associated with any factors without TIDI-fatigue also being associated.

When investigated individually sleepiness and fatigue were moderately associated (|β| between 0.27 and 0.58) with all mental health outcomes (see Supplementary Materials). Physical activity was significantly associated with fatigue with small standardized effect (β = -0.08 for fatigue) and sleepiness was not significantly related to level of physical activity.

## Discussion

In this paper we outlined the development and initial validation of a new integrated measure of sleepiness and fatigue, the TIDI. The eight-item measure comprises a sleepiness subscale and a fatigue subscale, which can be used individually or combined into a single tiredness measure. The subscales show convergent and divergent validity with existing sleepiness and fatigue measures, are simple to complete and score, have high internal reliability, and are context-independent (i.e. do not require participants to have been exposed to particular situations). In subsequent analysis the sleepiness and fatigue subscales were tested for specific relationships with sleep features – finding that sleepiness was more associated than fatigue with reduced sleep duration and increased apnoea risk score, versus fatigue being more associated than sleepiness with insomnia, napping, and nightmares. Assessing both subscales entered together against mental health measures revealed that fatigue (but not sleepiness) was associated with depression, anxiety, reduced wellbeing, and reduced physical activity, and that both fatigue and sleepiness have an association with psychotic experiences. The results support tiredness in general, and fatigue in particular, as a relevant factor to explore in mental health presentations.

The aim in developing this scale was to enable investigation of sleepiness and fatigue given the high and overlapping presentation of these symptoms. The TIDI successfully differentiates sleepiness and fatigue, as established by the final bifactor structure (ratified in an independent test sample), and supported by convergent and divergent validity tests against established sleepiness and fatigue measures. The value of this differentiation is demonstrated by comparing the general “tiredness” relationships with sleep and mental health outcomes (i.e. tiredness being significantly related to all outcome variables) to the subscale analyses which indicated many relationships with tiredness were in fact driven primarily by one domain (typically fatigue). In some cases these specific relationships were as expected based on the prior literature (e.g. sleepiness more strongly related to apnoea; fatigue more strongly related to insomnia) [11, 13]. However there were some unexpected results—e.g. no significant relationship between sleepiness and a desire to nap, which merit further exploration.

With respect to mental health outcomes, fatigue was more strongly related to increased severity of such difficulties than sleepiness in all cases. It is also worth noting indications of independent contributions of fatigue and sleepiness with psychotic experiences (paranoia and hallucinations). Overall the results support the relevance of fatigue to mental health presentations, though it has often been an overlooked feature [20]. Higher tiredness scores were reported by participants with a mental health diagnosis, and even higher scores amongst those with a diagnosis who were on a prescribed medication for their mental health concerns (compared to those not on medication). This is in addition to the direct relationships reported between tiredness and fatigue and all mental health outcomes. These results require validation in specific clinical groups (e.g. individuals diagnosed with depression), but they indicate it may be worthwhile to assess the effect of established sleep interventions on tiredness and fatigue or to assess the effect of treatments for anxiety or depression, for example, on tiredness and fatigue.

### Limitations

A limitation of the measure development process is that the item pool was only generated based on existing scales and clinical experience, and did not include lived experience input from those with fatigue or sleepiness related conditions. This means that lived-experience elements of sleepiness and fatigue that are not captured by existing measures are at risk of being overlooked in this new measure. Relatedly, the use of a general population sample means that the scale requires further validation in clinical populations. Given the potential breadth of use across multiple clinical populations spanning sleep (e.g. apnoea), mental health (e.g. depression), and physical health (e.g. chronic fatigue), validation of TIDI items and differentiation properties in this range of clinical groups is a clear next step.

While the study group was non-clinical, there was nevertheless an elevated mental health and sleep disturbance burden—for example, 26% reported being in receipt of psychiatric medication, compared to 12.5% of the general UK population [45]. These disparities are likely due to the recruitment method (i.e. those with related concerns being more likely to respond to study advertisements). With the participant group being recruited from social media advertisements, there was a risk of undetected non-genuine or bot responding, although safeguards were in place to mitigate this e.g. removing respondents with unrealistically short survey completion times.

Given the study is cross-sectional it is not possible to infer causal direction of any of the relationships identified—in many cases they are likely bidirectional. We are also unable to report on test–retest reliability for the measure. The results are also based on self-report, with no objective measures of sleep or activity being collected. Objective sleep measurements would be highly valuable, and may assist with differentiating further between fatigue and sleepiness (given that self-report of sleep duration and efficiency is typically less when sleep disorders are present) [46]. It is surprising that only small relationships with tiredness, fatigue, or sleepiness were identified with physical activity levels. This deserves further investigation, ideally utilizing objective measures of activity alongside self-reported measures as used here, given the limitations of self-report in this domain [47].

The participant population was also less ethnically diverse (>95% white or white British), and more highly educated (57% with at least an undergraduate degree) than the UK average (81.7% and 33.8% respectively based on UK 2021 Census) [48]. With respect to ethnicity this lack of diversity prevented testing for measurement invariance in this respect due to the low sample size, which should be addressed in future research. The measure was indicated to be invariant both with respect to gender and mental health status.

## Conclusion

In conclusion the TIDI shows an ability to discriminate sleepiness and fatigue with convergent and divergent validity against established self-report measures in a general population sample. Analysis of relationships with sleep and mental health variables supported expected differentiation (e.g. sleepiness associated with apnoea, fatigue with insomnia), with other results requiring further research (e.g. fatigue and sleepiness having independent relationships with psychotic experiences). The TIDI can be used for research purposes in non-clinical samples, and given the high prevalence of mental health concerns in the study population may also be applicable in mental health populations for research purposes. For clinical purposes the TIDI requires further validation in e.g. clinical sleep and physical health presentations where fatigue and/or sleepiness are common comorbidities or differential indicators, but shows promise in allowing measurement and disaggregation of these common and disabling symptoms.

## Supplementary Material

Supplementary_materials_zpag072(1)

## Data Availability

Data are available from the corresponding author upon request.
