# Supplementary material for: Development and validation of the tiredness identification index: a combined scale for the assessment of fatigue and sleepiness
Source: Sleep Adv. 2026 Jul 2;7(3):zpag072. doi: 10.1093/sleepadvances/zpag072 (PMC13446577; doi:10.1093/sleepadvances/zpag072)
Supplement: Supplementary file 1 [file supplementary_materials_zpag072(1).docx]

Supplementary materials

Development and validation of the Tiredness Identification Index (TIDI): a combined scale for the assessment of fatigue and sleepiness

Sarah Reeve^1,2^, Bryony Sheaves^3,4^, Bao Sheng Loe^5^, Daniel Freeman^3,4^

^1^ Norwich Medical School, University of East Anglia, Norwich, UK,

^2^Cambridgeshire and Peterborough NHS Foundation Trust, Cambridge, UK

^3^Department of Experimental Psychology, University of Oxford, UK

^4^Oxford Health NHS Foundation Trust, Oxford, UK.

^5^University of Cambridge, Cambridge, UK

Corresponding author

Sarah Reeve, [sarah.reeve@uea.ac.uk](mailto:sarah.reeve@uea.ac.uk)

Norwich Medical School, University of East Anglia, Norwich, UK, NR4 7TJ

Supplementary materials 1 – initial item pool

| This questionnaire assesses the impact of tiredness. In the last week, how much of an issue have you had with…. | | | | | |
| --- | --- | --- | --- | --- | --- |
| **(circle as appropriate)** | **0 (none)** | **1 (slight)** | **2 (moderate)** | **3 (very much)** | **4 (extreme)** |
| 1. **Feeling unable to concentrate** | 0 | 1 | 2 | 3 | 4 |
| 2. **Feeling sluggish** | 0 | 1 | 2 | 3 | 4 |
| 3. **Being easily irritated** | 0 | 1 | 2 | 3 | 4 |
| 4. **Feeling groggy** | 0 | 1 | 2 | 3 | 4 |
| 5. **Feeling that time is moving slower than normal** | 0 | 1 | 2 | 3 | 4 |
| 6. **Keeping eyes open** | 0 | 1 | 2 | 3 | 4 |
| 7. **Blinking more than usual** | 0 | 1 | 2 | 3 | 4 |
| 8. **Wanting to rest** | 0 | 1 | 2 | 3 | 4 |
| 9. **Being easily upset** | 0 | 1 | 2 | 3 | 4 |
| 10. **Feeling worn out** | 0 | 1 | 2 | 3 | 4 |
| 11. **Eyes feeling heavy** | 0 | 1 | 2 | 3 | 4 |
| 12. **Avoiding activity** | 0 | 1 | 2 | 3 | 4 |
| 13. **Avoiding social events** | 0 | 1 | 2 | 3 | 4 |
| 14. **Feeling weak** | 0 | 1 | 2 | 3 | 4 |
| 15. **Eyes feeling itchy or stingy** | 0 | 1 | 2 | 3 | 4 |
| 16. **Feeling dozy** | 0 | 1 | 2 | 3 | 4 |
| 17. **Feeling weary** | 0 | 1 | 2 | 3 | 4 |
| 18. **Slurring my words** | 0 | 1 | 2 | 3 | 4 |
| 19. **Feeling unable to cope** | 0 | 1 | 2 | 3 | 4 |
| 20. **Lacking motivation** | 0 | 1 | 2 | 3 | 4 |
| 21. **Feeling anxious** | 0 | 1 | 2 | 3 | 4 |
| 22. **Wanting to lie down** | 0 | 1 | 2 | 3 | 4 |
| 23. **Slowed down movements** | 0 | 1 | 2 | 3 | 4 |
| 24. **Feeling half-awake** | 0 | 1 | 2 | 3 | 4 |
| 25. **Nodding off** | 0 | 1 | 2 | 3 | 4 |
| 26. **Feeling frustrated** | 0 | 1 | 2 | 3 | 4 |
| 27. **Feeling sleepy** | 0 | 1 | 2 | 3 | 4 |
| 28. **Feeling low in energy** | 0 | 1 | 2 | 3 | 4 |
| 29. **Feeling achy** | 0 | 1 | 2 | 3 | 4 |
| 30. **Wanting to nap** | 0 | 1 | 2 | 3 | 4 |
| 31. **Avoiding work** | 0 | 1 | 2 | 3 | 4 |
| 32. **Feeling low or sad** | 0 | 1 | 2 | 3 | 4 |
| 33. **Slowed down thoughts** | 0 | 1 | 2 | 3 | 4 |
| 34. **Feeling fatigued** | 0 | 1 | 2 | 3 | 4 |
| 35. **Feeling exhausted** | 0 | 1 | 2 | 3 | 4 |
| 36. **Drifting off** | 0 | 1 | 2 | 3 | 4 |
| 37. **Feeling blank** | 0 | 1 | 2 | 3 | 4 |
| 38. **Yawning** | 0 | 1 | 2 | 3 | 4 |
| 39. **Feeling drained** | 0 | 1 | 2 | 3 | 4 |
| 40. **Feeling drowsy** | 0 | 1 | 2 | 3 | 4 |

**Supplementary Material 2 – individual item factor loadings**

Item loadings – two factor

| Item | Sleepiness | Fatigue |
| --- | --- | --- |
| **Keeping eyes open** | 0.728 |  |
| **Blinking more than usual** | 0.584 |  |
| **Drifting off** | 0.713 |  |
| **Wanting to nap** | 0.764 |  |
| **Feeling unable to concentrate** |  | 0.745 |
| **Feeling weak** |  | 0.766 |
| **Feeling low in energy** |  | 0.855 |
| **Feeling exhausted** |  | 0.877 |

Item loadings - bifactor

| Item | Sleepiness | Fatigue | General |
| --- | --- | --- | --- |
| **Keeping eyes open** | 0.370 |  | 0.746 |
| **Blinking more than usual** | 0.300 |  | 0.563 |
| **Drifting off** | 0.137 |  | 0.683 |
| **Wanting to nap** | -0.475 |  | 0.910 |
| **Feeling unable to concentrate** |  | 0.465 | 0.572 |
| **Feeling weak** |  | 0.521 | 0.563 |
| **Feeling low in energy** |  | 0.609 | 0.625 |
| **Feeling exhausted** |  | 0.544 | 0.676 |

**Supplementary material 3 – individual subscale analyses**

Fatigue subscale only

|  | **Predictor** | **Estimate (StdError)** | **Standardised estimate (StdError)** | **Statistic** | **p-value** |
| --- | --- | --- | --- | --- | --- |
| **Sleep** |  |  |  |  |  |
| Sleep duration (mins) | (Intercept) | 432.43 (2.53) | 0.00 (0.01) | -0.02 | 0.984 |
|  | **TIDI_f** | **-1.13 (0.3)** | **-0.05 (0.01)** | **-3.78** | **<0.001** |
| Sleep efficiency | (Intercept) | 0.86 (0.00) | 0.00 (0.01) | -0.10 | 0.922 |
|  | **TIDI_f** | **-0.01 (0.00)** | **-0.22 (0.01)** | **-18.36** | **<0.001** |
| Nap duration (mins) | (Intercept) | 29.9 (2.5) | -0.12 (0.02) | -5.22 | 0.000 |
|  | **TIDI_f** | **3.48 (0.25)** | **0.3 (0.02)** | **13.74** | **<0.001** |
| Insomnia Severity Index (ISI) | (Intercept) | 12.65 (0.13) | 0.00 (0.01) | -0.19 | 0.846 |
|  | **TIDI_f** | **0.95 (0.02)** | **0.61 (0.01)** | **62.76** | **<0.001** |
| Nightmares (n over previous two weeks) | (Intercept) | 0.33 (0.08) | 0.00 (0.01) | -0.12 | 0.905 |
|  | **TIDI_f** | **0.20 (0.01)** | **0.28 (0.01)** | **22.59** | **<0.001** |
| STOP-BANG | (Intercept) | 1.03 (0.05) | 0.01 (0.02) | 0.53 | 0.598 |
|  | **TIDI_f** | **0.06 (0.01)** | **0.16 (0.02)** | **10.31** | **<0.001** |
| **Mental health** |  |  |  |  |  |
| Depression (DASS) | (Intercept) | 7.78 (0.12) | 0.00 (0.01) | -0.33 | 0.738 |
|  | **TIDI_f** | **0.80 (0.01)** | **0.58 (0.01)** | **55.19** | **<0.001** |
| Anxiety (DASS) | (Intercept) | 7.95 (0.09) | 0.00 (0.01) | -0.28 | 0.781 |
|  | **TIDI_f** | **0.5 (0.01)** | **0.52 (0.01)** | **47.48** | **<0.001** |
| Paranoia (SPEQ) | (Intercept) | 18.13 (0.34) | 0.00 (0.01) | -0.12 | 0.905 |
|  | **TIDI_f** | **1.29 (0.04)** | **0.38 (0.01)** | **31.79** | **<0.001** |
| Hallucinations (SPEQ) | (Intercept) | 0.38 (0.15) | 0.00 (0.01) | -0.12 | 0.908 |
|  | **TIDI_f** | **0.48 (0.02)** | **0.33 (0.01)** | **27.01** | **<0.001** |
| Wellbeing (WEMWBS) | (Intercept) | 53.99 (0.24) | 0.00 (0.01) | 0.18 | 0.856 |
|  | **TIDI_f** | **-1.48 (0.03)** | **-0.56 (0.01)** | **-52.12** | **<0.001** |
| Physical activity (IPAQ) | (Intercept) | 2985.35 (76.54) | 0.00 (0.01) | 0.02 | 0.985 |
|  | **TIDI_f** | **-54.02 (9.04)** | **-0.08 (0.01)** | **-5.98** | **<0.001** |

Sleepiness subscale only

|  | **Predictor** | **Estimate (StdError)** | **Standardised estimate (StdError)** | **Statistic** | **p-value** |
| --- | --- | --- | --- | --- | --- |
| **Sleep** |  |  |  |  |  |
| Sleep duration (mins) | (Intercept) | 12.10 (0.09) | 0.00 (0.01) | -0.02 | 0.985 |
|  | **TIDI_s** | **-1.92 (0.35)** | **-0.07 (0.01)** | **-5.56** | **<0.001** |
| Sleep efficiency | (Intercept) | 0.83 (0.00) | 0.00 (0.01) | -0.04 | 0.970 |
|  | **TIDI_s** | **-0.01 (0.00)** | **-0.13 (0.01)** | **-10.93** | **<0.001** |
| Nap duration (mins) | (Intercept) | 40.52 (2.64) | -0.12 (0.03) | -4.59 | 0.000 |
|  | **TIDI_s** | **2.70 (0.31)** | **0.2 (0.02)** | **8.60** | **<0.001** |
| Insomnia Severity Index (ISI) | (Intercept) | 15.08 (0.13) | 0.00 (0.01) | -0.07 | 0.948 |
|  | **TIDI_s** | **0.81 (0.02)** | **0.45 (0.01)** | **41.45** | **<0.001** |
| Nightmares (n over previous two weeks) | (Intercept) | 0.94 (0.07) | 0.00 (0.01) | 0.01 | 0.991 |
|  | **TIDI_s** | **0.15 (0.01)** | **0.19 (0.01)** | **14.86** | **<0.001** |
| STOP-BANG | (Intercept) | 0.90 (0.04) | 0.01 (0.02) | 0.85 | 0.396 |
|  | **TIDI_s** | **0.1 (0.01)** | **0.24 (0.02)** | **15.65** | **<0.001** |
| **Mental health** |  |  |  |  |  |
| Depression (DASS) | (Intercept) | 10.51 (0.13) | 0.00 (0.01) | 0.08 | 0.934 |
|  | **TIDI_s** | **0.58 (0.02)** | **0.36 (0.01)** | **30.16** | **<0.001** |
| Anxiety (DASS) | (Intercept) | 9.43 (0.09) | 0.00 (0.01) | 0.09 | 0.932 |
|  | **TIDI_s** | **0.40 (0.01)** | **0.36 (0.01)** | **30.04** | **<0.001** |
| Paranoia (SPEQ) | (Intercept) | 21.48 (0.32) | 0.00 (0.01) | 0.10 | 0.921 |
|  | **TIDI_s** | **1.1 (0.05)** | **0.28 (0.01)** | **22.80** | **<0.001** |
| Hallucinations (SPEQ) | (Intercept) | 1.34 (0.14) | 0.00 (0.01) | 0.11 | 0.910 |
|  | **TIDI_s** | **0.46 (0.02)** | **0.27 (0.01)** | **22.23** | **<0.001** |
| Wellbeing (WEMWBS) | (Intercept) | 49.13 (0.25) | 0.00 (0.01) | -0.10 | 0.919 |
|  | **TIDI_s** | **-1.09 (0.04)** | **-0.36 (0.01)** | **-29.60** | **<0.001** |
| Physical activity (IPAQ) | (Intercept) | 2677.66 (69.4) | 0.00 (0.01) | -0.01 | 0.992 |
|  | **TIDI_s** | **-16.89 (10.40)** | **-0.02 (0.01)** | **-1.62** | **0.104** |

**Tiredness Identification Index**

| **This questionnaire assesses the impact of tiredness. In the last week, how much of an issue have you had with….** | | | | | |  |
| --- | --- | --- | --- | --- | --- | --- |
|  | **0**  **(none)** | **1**  **(slight)** | **2**  **(moderate)** | **3**  **(very much)** | **4**  **(extreme)** | |
| 1. **Feeling unable to concentrate** | 0 | 1 | 2 | 3 | 4 | |
| 2. **Keeping eyes open** | 0 | 1 | 2 | 3 | 4 | |
| 3. **Blinking more than usual** | 0 | 1 | 2 | 3 | 4 | |
| 4. **Feeling weak** | 0 | 1 | 2 | 3 | 4 | |
| 5. **Feeling low in energy** | 0 | 1 | 2 | 3 | 4 | |
| 6. **Wanting to nap** | 0 | 1 | 2 | 3 | 4 | |
| 7. **Drifting off** | 0 | 1 | 2 | 3 | 4 | |
| 8. **Feeling exhausted** | 0 | 1 | 2 | 3 | 4 | |

*Items 2, 3, 6, 7 = TIDI-s; items 1, 4, 5, 8 = TIDI-f*
